# Analysing the Floral Elements of the Lost Tree of Easter Island: A Morphometric Comparison between the Remaining *Ex-Situ* Lines of the Endemic Extinct Species *Sophora toromiro*

**DOI:** 10.1371/journal.pone.0115548

**Published:** 2014-12-19

**Authors:** Thomas A. Püschel, Jaime Espejo, María-José Sanzana, Hugo A. Benítez

**Affiliations:** 1 Faculty of Life Sciences, University of Manchester, Manchester, United Kingdom; 2 Instituto de Alta Investigación, Universidad de Tarapacá, Arica, Chile; 3 Facultad de Cs. Forestales, Programa postgrado Universidad de Concepción, Concepción, Chile; 4 Departamento de Zoología, Facultad de Ciencias Naturales y Oceanográficas, Universidad de Concepción, Concepción, Chile; University of Delhi, India

## Abstract

*Sophora toromiro* (Phil) Skottsb. is a species that has been extinct in its natural habitat Easter Island (Rapa Nui) for over 50 years. However, seed collections carried out before its extinction have allowed its persistence *ex-situ* in different botanical gardens and private collections around the world. The progenies of these diverse collections have been classified in different lines, most of them exhibiting high similarity as corroborated by molecular markers. In spite of this resemblance observed between the different lines, one of them (Titze) has dissimilar floral elements, thus generating doubts regarding its species classification. The floral elements (wing, standard and keel) belonging to three different *S. toromiro* lines and two related species were analyzed using geometric morphometrics. This method was applied in order to quantify the floral shape variation of the standard, wing, and keel between the different lines and control species. Geometric morphometrics analyses were able to distinguish the floral elements at both intra (lines) and inter-specific levels. The present results are on line with the cumulative evidence that supports the Titze line as not being a proper member of the *S. toromiro* species, but probably a hybridization product or even another species of the Edwardsia section. The reintroduction programs of *S. toromiro* should consider this information when assessing the authenticity and origin of the lines that will be used to repopulate the island.

## Introduction

*Sophora toromiro* (Phil.) Skottsb. is a shrub or small tree (height: 2 meters; diameter: 10–15 cm) characterized by its twisted trunk, several branches and longitudinally fissured reddish-brown bark (leaves 2.5–5.0 cm, leaflets 7–21 mm narrowly elliptic, oppositive to subopposite 9–12 mm×4–7 mm. Its flowers are hermaphrodite 1.5–3.0 cm long with a solitary yellow corolla) [1], [2]. It is a flowering tree of the legume family Fabaceae that is endemic to Easter Island or Rapa Nui. It belongs to the ‘Edwardsia’ section which is composed by over 20 species distributed in the South Pacific [3], [4]. These native flora inhabiting oceanic islands are highly interesting due to their particular evolutionary history and endemism [5], [6]. Additionally, what makes *S. toromiro* even more remarkable is the fact that it became extinct relatively recently, although it survived *ex-situ* due to some seed collections carried out by some explorers.

Rapa Nui is a special territory of Chile that was annexed in 1888. This island is located 3,765 km west of continental Chile. The fragility of this insular ecosystem, along with the dramatic human intervention of the island experienced for over 500 years, have shaped a remarkable scenario to study the impact of human populations on the ecology of insular environments [7]–[10]. The decline of the Rapa Nui culture during the XVIII century is still a highly investigated period in the history of this island [11]–[14]. Despite the divergent opinions regarding the underlying causes of this phenomenon, most of the evidence suggests deforestation of the island [12]. These changes in the landscape composition have been corroborated by several paleobotanic studies [15], [16]. The demographic growth and the intensive use of land probably were main factors affecting the distribution and number of vegetal species in the island. Several pollen records have shown that before the arrival of the Polynesians ca. 880–1200 A.D. the island had a richer and more diverse flora [14]–[17]. Furthermore, the records have shown an increased use of fire for both clearing and agriculture along with the first human settlements [18], [19]. Archaeological surveys have suggested an intensive use of land to support the growing population [10]. The *S. toromiro* tree had a special importance for the Rapa Nui native population according to the chronicles written by the first European explorers that visited the island [20]. Increasing archaeological evidence actually demonstrates that *S. toromiro* was used as firewood and as raw material for the manufacture of ritual objects [21]–[23]. The scarce literature of that time has suggested that *S. toromiro* could have endured in its native habitat until the end of the XIX century, when the island was exploited and colonized by a company authorized by the Chilean state (1895–1953). The company imposed an extreme regime for the native species, especially due to the introduction of thousands of sheep. Toro [24] described that at the end of the XIX century there were over 18,000 sheep in the island, while in the ‘50s their population have increased to around 40,000. This intense livestock exploitation seriously affected Rapa Nui's flora, transforming its landscape into a meadow type, dominated by strata of grasses and sedges as seen nowadays. It has been noticed that the current landscape of Easter Island is characterized by only 7.7% of endemic species [25]–[28].

*S. toromiro* has been recognized as extinct in the wild for over fifty years, since its last exemplar was felled in the inner hillside of the Rano Kao volcanic crater. However, some seed collections carried out during the ‘50s allowed its persistence *ex-situ*, although all the reintroduction attempts performed since 1965 have failed. The existing progenies of *S. toromiro* are product of its self-pollinating mechanism, which has generated seeds and plants that are protected in botanic gardens and private collections mainly in Europe and Chile. Maunder et al. [29] and Maunder et al. [30] classify these specimens in “lines” and to this date there are around 13 of them. Nevertheless, the only documented seed sources correspond to those collected by Efrain Volosky who cultivated them in the National Botanic Garden of Viña del Mar and Thor Heyerdahl in 1956, who donated them to the Botanic Garden of Göteborg [31]. The available literature also mentioned seed collections carried out by Gana [32] and Lavanchery [33] but there is no evidence or traceability of these germplasms [20]. Due to this reason, it was decided to analyse the Göteborg and Viña del Mar lines because they certainly correspond to progenies derived from *S. toromiro* specimens from Rapa Nui. In addition, samples of the Titze line were collected due to their importance in the reintroduction attempts carried out by the Chilean government. This specific line does not have a proper historical record and it has been cultivated for years in the nursery of Pablo Titze in Talagante, Chile [30]. It is important to analyse this line because it is by far the most common *S. toromiro* line found in Chile (it is cultivated in several private gardens) and because seeds belonging to this line were donated to the Instituto Forestal (INFOR), Chile. This institution started cultivating this particular progeny in order to donate the resulting plants to Eastern Island for a future reintroduction attempt. Nonetheless, as we discussed below there are serious concerns regarding the taxonomic status of this line. Specimens of this particular progeny derived from the Titze line were included in the analysis under the name of INFOR.

The *Sophora spp.* flower is singular due to its shape, known as *papilioinides* (because its shape resembles the insects of the order Lepidoptera). The floral structure of *Sophora spp.* and the ‘Edwardsia’ section is considered a successful breeding strategy mechanism [34], being visited by both insects and birds [35]–[37]. The flower elements are zygomorphic, hence the standard is clearly distinct from the other petal elements (i.e. the wing and keel) [37]. The standard is the part of the corolla that covers and protects the rest of the floral and breeding structures during the floral blossom stage [38]. The ontogeny of these parts shows that the keel is the first structure to be developed, while the standard is the last one. Tucker [37] has pointed out that the standard has bilateral symmetry, while the wing and keel develop asymmetrically. Reiche [39] in the “Tratado de Flora de Chile” describes the floral structures of *Sophora spp.*: “the standard is widely transoval or orbicular, commonly shorter than the keel. Oblong, oblique wings, longer than the keel (sic)”.

Based on some morphological differences observed in the leaflets of trees corresponding to different lines, Espejo et al. [40] have argued that the progenies of the Titze line could correspond to a hybrid between *S. toromiro* and *S. fernandeziana* or *S. cassioides* according to the observations of Ricci and Eaton [41]. This particular line was originally cultivated next to two specimens of the other mentioned species, and due to the similar flowering patterns between different *Sophora*'s species, it has been argued that they perhaps hybridized generating as result the Titze line. The suspicions increase when taking into account that this line does not have any clear historical records. Nonetheless, all the molecular studies that have compared the different lines have shown that they are highly similar [29], [30]. Chromosomic and cytometric studies have also exhibited high levels of similarity (in preparation), hence the doubt persists. In the present study we try to differentiate the floral structure between different *S.toromiro* lines by applying geometric morphometrics. Basically, we try to determine if the qualitative morphological differences observed between lines are robust enough to be distinguished by applying quantitative techniques. This is highly relevant due to the conservation status of this species, as well as for future re-introduction attempts.

The structural elements of flowers often show high complexity and the arrangement of petals or leaves often exhibit intricate symmetry. Therefore, floral traits are inherently multivariate and variation should be assessed using appropriate morphometric methods. Morphometrics is used routinely to address a wide range of problems in plant ecology, evolutionary and developmental biology. For instance, several applications have been performed in taxonomy or phylogeny e.g. [42,43,44], ecology [45]–[47], natural selection [47]–[52], evo-devo [53], symmetry and allometry [53]–[55]. Geometric morphometrics was preferred in this study based on its coherent statistical foundations and its capacity to distinguish subtle differences [44], [50], [56]–[58]. Due to the fact that the morphological differences between the floral elements of the different lines are subtle, geometric morphometrics is suitable to assess these minimal dissimilarities. Based on these background data, it was tested if the Titze line belongs or not to the *S. toromiro* species. Assuming that the genotypes generated via self-pollination are stable in this species, and taking into the account that the studied lines do have a high phenotypic homogeneity, it is logical to wonder why the floral elements of the Titze line look so different when compared to the other remaining progenies.

## Material and Methods

### Ethics Statement

Our samples did not need any specific permission for collection (locations/activities) and this study did not involve any endangered or protected species (the species used are wildly extinct, there are remains only in botanic gardens).

### Data Collection

Flowers were collected from *S. toromiro* specimens belonging to the Göteborg (Got), Botanic Garden of Viña del Mar and Titze lines Maunder et al. [29]. The specimens classified as INFOR correspond to descendants of the Titze line, although they come from the Instituto Forestal, Chile. They were classified separately in order to analyze if they are similar or not to its parental line. Two other species were included as outgroups: *Sophora cassioides* (Phil) Sparre., and *Sophora macrocarpa* Sm. They are taxonomically related with *S. toromiro* and were collected in the Rucamanqui farm (37° 15′ S, 71° 55′ W, height 485 m) Chile. All the germoplasms included were cultivated in greenhouses belonging to Forestal Mininco S.A. under the same environmental conditions, and located in the city of Los Ángeles, Chile.

**Figure 1:**
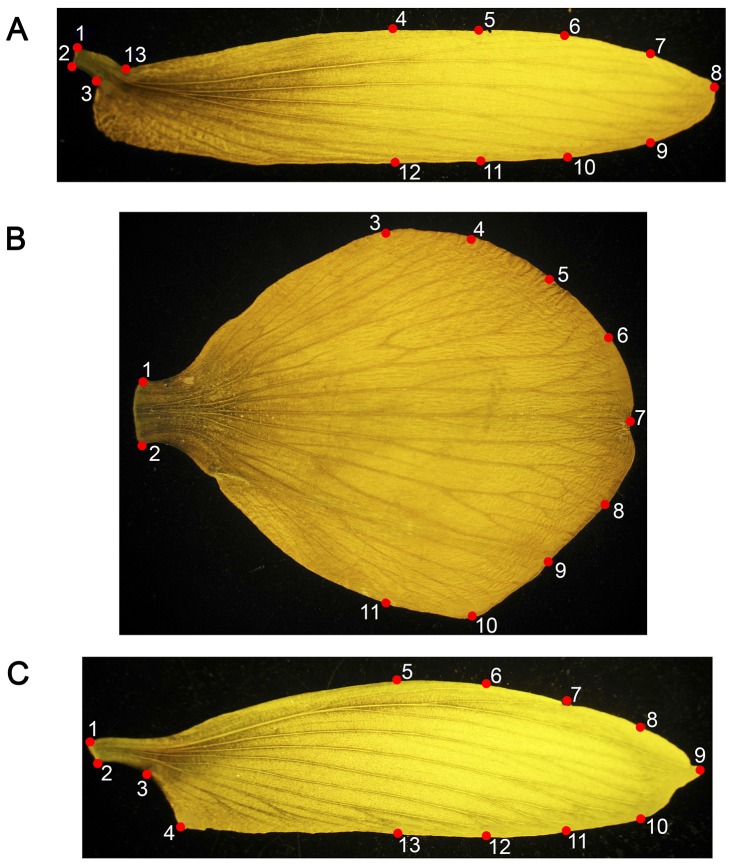
Homologous landmark map of the floral elements. a) wing 13 landmarks, b) standard 11 landmarks c) keel 13 landmarks.

The flowers were collected in the pre-anthesis stage; hence they were almost a complete flower even though they had not opened yet the standard or the wings (Fig 1). At that stage the stamen was barely visible, although the pistil was clearly evident. The number of flowers and blooming trees are shown in Table 1.

**Table 1:** Number of floral structures classified by origin.

Origen/Structure N	Standard	Wing	Keel
Göteborg (Got)	16	18	17
INFOR	4	5	5
Jardín Botánico Viña (JBV)	15	18	18
Titze (Tit)	33	31	31
*Sophora macrocarpa* (Sm)	12	12	12
*Sophora cassioides* (Sc)	10	10	8

* The flowers of two taxonomically related species were included as outgroups: *Sophora cassioides* and *Sophora macrocarpa*.

The samples were dried and pressed at ambient temperature during 72 hours. Then, they were photographed using a Cannon 620 machine mounted on a Zeiss 3MB (6,4x) magnifying glass and focused fibre-optic light lamps. Homologous morphological landmarks were defined for each floral element.

### Geometric Morphometrics and multivariate analysis of floral elements

Geometric morphometrics was applied in order to quantify the shape variation of the flower elements (standard, wing, and keel) between the different lines. 11 homologous landmarks (LMs) were digitized for the standard (90 samples), 13 for the wing (94 samples) and 13 for the keel (91 samples) on single images of each leaf using the tpsDig software v.2.17 [59] (Fig. 1) (S1 Table). 2D Cartesian coordinates were obtained for all landmarks and the shape information was extracted using a full Procrustes fit [60], [61] (S2 Table). Procrustes superimposition is a procedure that removes the information of size, position and orientation in order to obtain shape variables [60]. A principal component analysis (PCA) was carried out to quantify the shape variation associated with each shape dimension. The inter-location differences among floral elements were analysed using a canonical variate analysis (CVA). The differences between the different lines were assessed by applying a Procustes ANOVA. All the aforementioned analyses were performed using MorphoJ v1.05d [62]. Finally, an UPGMA of the Procrustes distances between the average shapes of the analyzed lines was computed for each one of the structures using R v. 3.0.3 (www.R-project.org) to visualize the morphological affinities between lines.

## Results

**Figure 2:**
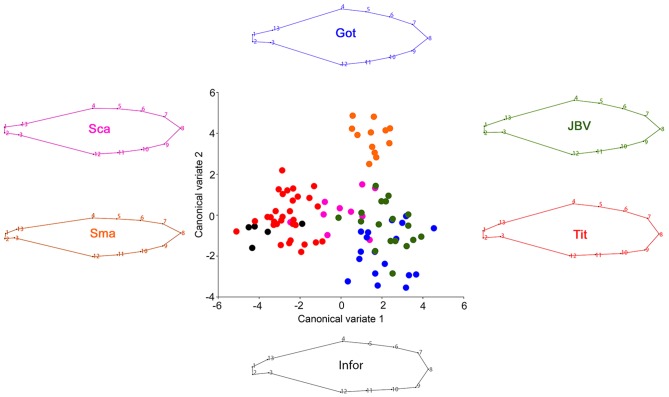
Canonical variate analysis of the wing comparing the different *Sophora toromiro* lines and the two out-groups, Goteborg (Got), INFOR, Jardin Botanico Viña (JBV), Titze (Tit), *Sophora macrocarpa* (Sma), *Sophora cassioides* (Sca). The wireframe represents the average shape of each one of the analyzed lines.

The three floral structures were differentiated according to the relative displacement of specific landmarks. The wing was mostly distinguished by the particular displacement of landmarks #1, #2, #3 (mostly basal coordinates) and the landmark #8 (apical point), Fig 2.

**Figure 3:**
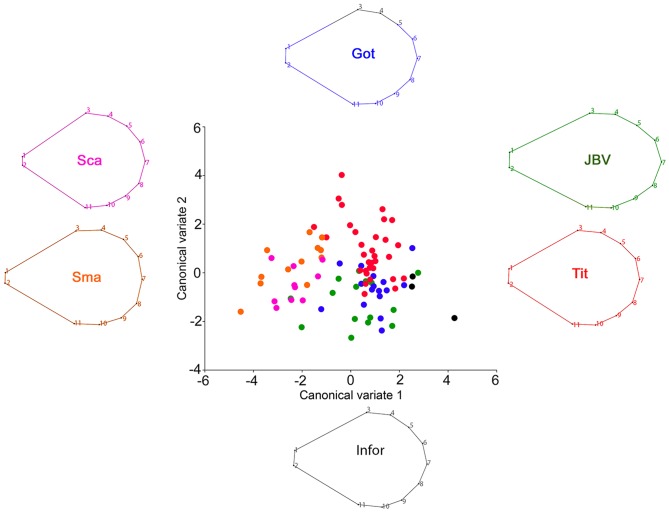
Canonical variate analysis of the standard comparing the different *Sophora toromiro* lines and the two out-groups, Goteborg (Got), INFOR, Jardin Botanico Viña (JBV), Titze (Tit), *Sophora macrocarpa* (Sma), *Sophora cassioides* (Sca). The wireframe represents the average shape of each one of the analyzed lines.

**Figure 4:**
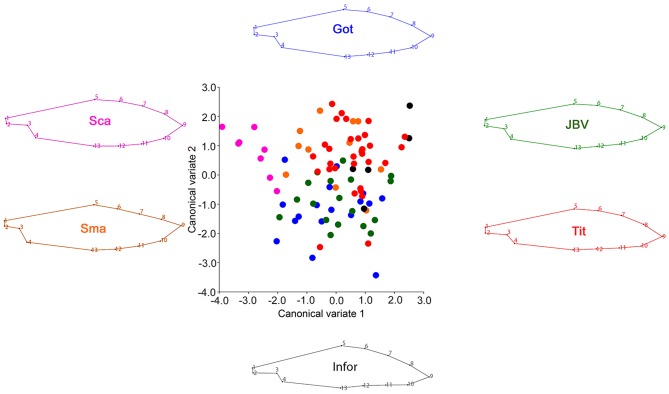
Canonical variate analysis of the keel comparing the different *Sophora toromiro* lines and the two out-groups, Goteborg (Got), INFOR, Jardin Botanico Viña (JBV), Titze (Tit), *Sophora macrocarpa* (Sma), *Sophora cassioides* (Sca). The wireframe represents the average shape of each one of the analyzed lines.

The standard was differentiated by the relative displacement of the landmarks #1, #2 (at the base) and the symmetric landmarks #3, #11 and #5, #9 (margins of the wing), Fig 3. While the keel showed a relative displacement of the landmarks #2, #3, #4 (base area) and the landmark #9 (apical point), Fig 4.

The PCA of the three floral structures showed that the first three PCs accounted for more than 70% of the total shape variation, thus providing a reasonable approximation of the total amount of flower shape variation. The first two floral structures were mostly differentiated by the first PCA (Standard: PC1+PC2+PC3 = 53.6%, 12.58% and 8.28 & Wing: PC1+PC2+PC3 = 56.65%, 14.79% and 10.44%), although the keel showed a more distributed variation visible in the first two PCs (Keel: PC1+PC2+PC3 = 38.96%, 29.17% and 12.68%).

The CVA showed a significant differentiation among lines and the other *Sophora spp.* based on the Procrustes distances. From a descriptive perspective the anatomical changes associated with the two CVA axes were as follows: a) wing (Fig 2.): CV1 characterizes a central broadening of the petal and a relative displacement of the midpoint tip of the apical petal portion (landmark 8), whilst CV2 shows a global expansion of the petal structure; a) standard (Fig 3.): CV1 is related to a relative expansion of the superior and inferior margins with a contraction of the petal tip, while CV2 is associated with an elongation of the central part of the petal along a supero-inferior plane; c) keel (Fig. 4.): CV1 is associated with a global contraction of this petal structure, whereas CV2 is mostly related to an extreme elongation of the petal origin and a relative contraction of its inferior margin. It is important to cautiously consider the above descriptions, because they are based on a CVA. CVs are aligned with the major axes of variation among groups, therefore they account for the maximum amount of among-group variance relative to within-group variance, which not necessarily reflects the actual morphological differences between groups. Finally, a Procrustes ANOVA of the flower structure shapes showed highly significant differences between the lines as well, P<0.0001 (Table 2), showing a clear statistical differentiation.

**Table 2:** Procrustes ANOVA for both centroid size and shape of the *Sophora* lines.

Centroid size							
Effect	SS	MS	df	F	P	Pillai tr.	P(param)
**Standard**	0.000062	0.000012	5	1.81	0.12		
Individual	0.000577	0.000007	84				
**Wing**	0.000038	0.000008	5	6.99	<.0001		
Individual	0.000096	0.000001	88				
**Keel**	0.000045	0.000009	5	6.16	<.0001		
Individual	0.000124	0.000001	85				
**Shape**							
**Standard**	0.08414393	0.000934933	90	3.42	<.0001	1.62	<.0001
Individual	0.41389707	0.000273741	1512	NaN	NaN		
**Wing**	0.11651277	0.001059207	110	9.94	<.0001	2.38	<.0001
Individual	0.20629406	0.000106557	1936	NaN	NaN		
**Keel**	0.06369427	0.000579039	110	3.66	<.0001	2.17	<.0001
Individual	0.29611693	0.000158351	1870	NaN	NaN		

Sums of squares (SS) and mean squares (MS) are in units of Procrustes distances (dimensionless).

**Figure 5:**
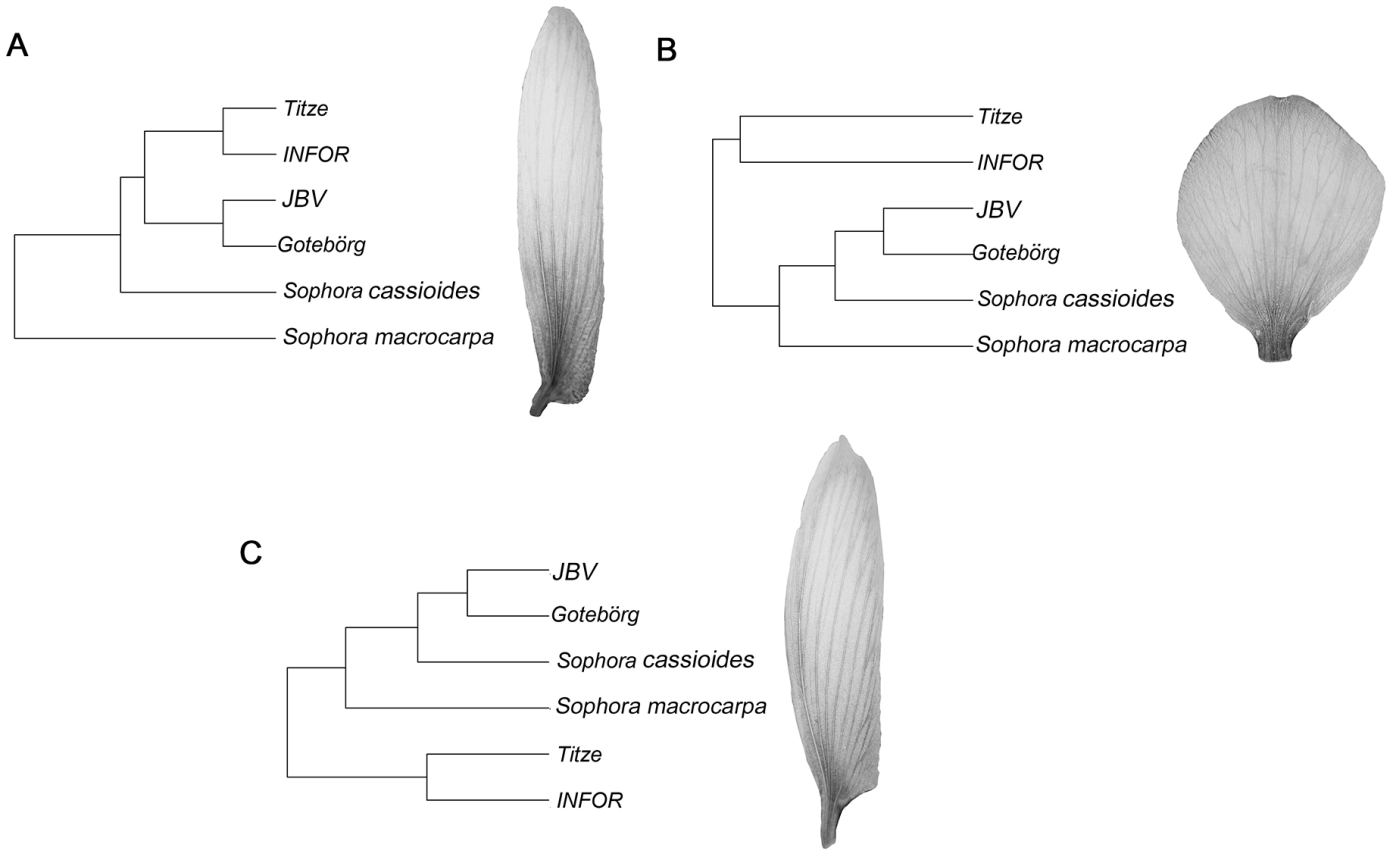
UPGMA of the procrustes distances between the average shapes of the different lines: a) wing, b) standard and c) keel.

The UPGMA (Fig.5) of the Procrustes distances between the average shapes of the lines showed a relatively consistent scenario. Both the standard b) and the keel c) clustered JBV and Göteborg together as expected for the same species, being closely related (in morphological terms) with *S. cassioides* and *S. macrocarpa*. On the other hand, the Titze line stood out as an out-group, being more different with respect to the *S. toromiro* specimens than the control species. The wing a) showed similar relationships, however the group formed by JBV and Göteborg was closer to the Titze group than the control species.

## Discussion

The present work has shown the usefulness of geometric morphometrics as a quantitative tool to characterize floral structures. This is highly relevant because flower shape variation in angiosperms has been related to several ecological factors such as breeding strategies of the pollinator/plant interaction [49], [63], [64]. Interestingly, geometric morphometrics was able to distinguish between the different lines, showing that the progenies of the Titze line are notoriously different with respect to rest, as it has been traditionally suggested by Espejo et al. (2008). This is remarkable since the first mention of this species as *Edwardsia toromiro* done by Philippi [65] and the first valid description of *Sophora toromiro* by Skottsberg [1] do not describe the flower at all. The work of Rodríguez et al. [66] used only herbarium specimens while the study of Mackinder and Staniforth [2] solely analyzed samples belonging to an *ex-situ* specimen from the Botanisher Garten Bonn. It has been shown previously that geometric morphometrics is a useful tool to differentiate taxa of complex groups when conventional systematic techniques yield poor results [44], [67], [68].

In current literature, Heenan et al. [4] states that the Edwardsia section has a high similarity between species (ca. 99%–100%) using rbcL and ITS, which discourages any intra-specific comparison within the Edwardsia section using these techniques. This is unsurprising because endemic island congeners often display high similarity, but also often with strong morphological differences.

It is important to establish if the lines are the same species, because future studies and conservational efforts depend on this. For instance, different reports such as Ricci and Eaton [41] using isoenzymatic systems and Maunder et al. [30] using RAPD and ISSR defined the Titze line within the *S. toromiro* species. Furthermore, Maunder et al. [30] differentiated not only the Titze line but also the Göteborg from Jardín Botánico progenies (although these lines have a well-known historical record that establishes their adscription to the *S. toromiro* species). The results here presented do not support this latter division, showing on one hand that these two progenies have a high resemblance at the phenotypic level, and that the Titze line has noticeably different floral structures. It is intriguing that the SSR and RAPD studies of Maunder et al. (1999 and 2000) showed Titze closer to *S. toromiro*.

Regarding the discriminatory capability of each one of the floral elements, the canonical variate analysis of the floral structure of *S. toromiro* showed that the wing was the best element to differentiate between lines, as well as at the inter-specific level. The species *S. cassioides* and *S. macrocarpa* included as out-group [3], [4], were clearly separated from the *S. toromiro* lines when the wing was compared. Furthermore, not only the species were distinguished but also the Titze line was notoriously differentiated from the Göteborg/Jardín Botánico de Viña del Mar lines. The analysis of the keel showed that this structure exhibits a greater variation; nonetheless the three species were distinguished as well. However, all the different lines clustered relatively closely, without any clear separation. Finally, the standard also showed a clear separation between species, and a slight distinction between the Titze and the Göteborg/Jardín Botánico.

Interestingly, the UPGMA showed that the lines belonging to well-known *S. toromiro* specimens (i.e. Jardín Botánico de Viña del Mar and Göteborg) have higher morphological affinities with the control species (*S. cassioides* and *S. macrocarpa*) than the Titze line. Only the wing clustered more closely the Titze line with the *S. toromiro* individuals, nonetheless Jardín Botánico de Viña del Mar and Göteborg were still more morphologically similar. Additionally, the Titze line always clustered closer to the INFOR specimens, showing a similarity level expected for a parental line and its descendant.

Hybridization is a common phenomenon between species grouped in the Edwardsia section. Heenan et al. [34] reported at least six combinations between species in New Zealand, attributing this situation to the fact that some pollination vectors such as birds, exhibited a flower overlap between species that ultimately breaks down geographic barriers. In Chile this phenomenon has been reported by Donoso [69] between *S. macrocarpa* and *S. cassioides*.

The results here presented are expected to contribute to a better insight when assessing floral structure differences between highly similar progenies such as the *S. toromiro* lines. Although the Titze line was distinguished, it was not possible to determine the origin of this line or the parental species giving rise to this germoplasm, hence further analyses are required.

## Supporting Information

S1 Table Description of the landmarks on the floral elements.
(XLS)

S2 Table Raw Coordinate values for the different flower structures and their corresponding classifiers.
(XLSX)
